# Adolescents and Young Adults’ Sources of Contraceptive Information

**DOI:** 10.1001/jamanetworkopen.2024.33310

**Published:** 2024-09-13

**Authors:** Elizabeth Pleasants, Brooke Whitfield, Zoe H. Pleasure, Ci’erra Larsen, Dana Johnson, Riley J. Steiner, Laura D. Lindberg

**Affiliations:** 1 University of California Berkeley; 2 The University of Texas at Austin; 3 University of Washington School of Public Health, Seattle; 4 Power to Decide, Washington, DC; 5 Rutgers School of Public Health, Newark, New Jersey

## Abstract

This cross-sectional study examines whether there is an association between adolescents and young adults’ preferred and actual sources of contraceptive information and reporting sufficient contraceptive information.

## Introduction

Access to accurate contraceptive information is crucial for adolescents and young adults’ (AYA) sexual and reproductive well-being, fostering informed decision-making and bodily autonomy.^1^ Inadequate education and knowledge are associated with misperceptions of method risks, improper use, and discontinuation.^2,3,4^

AYAs’ contraceptive information sources vary, along with their quality and acceptability.^3,5^ With inequitable access to school-based sex education, online misinformation, and increasing health care restrictions,^1,2,5,6^ understanding AYAs’ receipt of contraceptive information is crucial. This study examines AYAs’ preferred and actual sources of contraceptive information and assesses associations with reporting sufficient contraceptive information.

## Methods

This cross-sectional study used deidentified secondary data and was exempt from institutional review; the original survey design and data collection were reviewed and approved by the Biomedical Research Alliance of New York institutional review board. This report followed the Strengthening the Reporting of Observational Studies in Epidemiology (STROBE) reporting guideline.

We used self-reported data from the 2023 Thanks, Birth Control Survey, administered online via Ipsos KnowledgePanel to AYAs aged 15 to 29 years assigned female at birth (AFAB). Minors were recruited through a parent, and all participants provided information consent or assent electronically. Fifty-three percent of participants reported ever having penile-vaginal sex (sample overview in eAppendix in Supplement 1). Participants reported preferred source(s) of contraceptive information (preferred sources) and their past-year contraceptive information source(s) (actual sources).

We examined the distribution of preferred and actual sources and conducted multivariable logistic regression to evaluate associations between participants reporting sufficient information to decide which contraceptive method was right for them (“sufficient information,” yes vs no/not sure) and actual information sources. Models were stratified by age (<18 years, 19-24 years, or 25-29 years) and adjusted for ever having sex. All analyses were conducted from February to June 2024 in Stata version 18.0 (StataCorp) using probability weights to represent the US population’s age, sex, race, census region, metropolitan status, and household income. Two-sided *P* < .05 was considered statistically significant.

## Results

Among a total of 1150 survey participants, 21% were aged less than 18 years, 44% were 18 to 24 years, and 35% were 25 to 29 years; 14% were Black, 24% were Hispanic, 51% were White, 6% were multiracial, and 5% were non-Hispanic other (race self-reported via survey). There were discrepancies between AYAs’ preferred and actual sources of contraceptive information and variations by age (Figure). Clinicians were the most commonly preferred source (68% among <18 years, 84% among 18-24 years, 87% among 25-29 years), but less common as actual sources (33% among  <18 years, 43% among 18-24 years, 50% among 25-29 years). More older participants reported websites as a preferred than actual source (36% vs 18% among 18-24 years, 38% vs 17% among 25-29 years). Although 59% of participants younger than 18 reported parents as a preferred source, only 36% received information from parents. Social networking sites, though less commonly preferred (6% among <18 years, 12% among 18-24 years, and 10% among 25-29 years), were the second-most common actual source for respondents aged 18 years or older (28% among 18-24 years, 18% among 25-29 years). Approximately one-third of participants did not receive contraceptive information in the past year (30% among <18 years, 35% among 18-24 years, 36% among 25-29 years).

**Figure.**
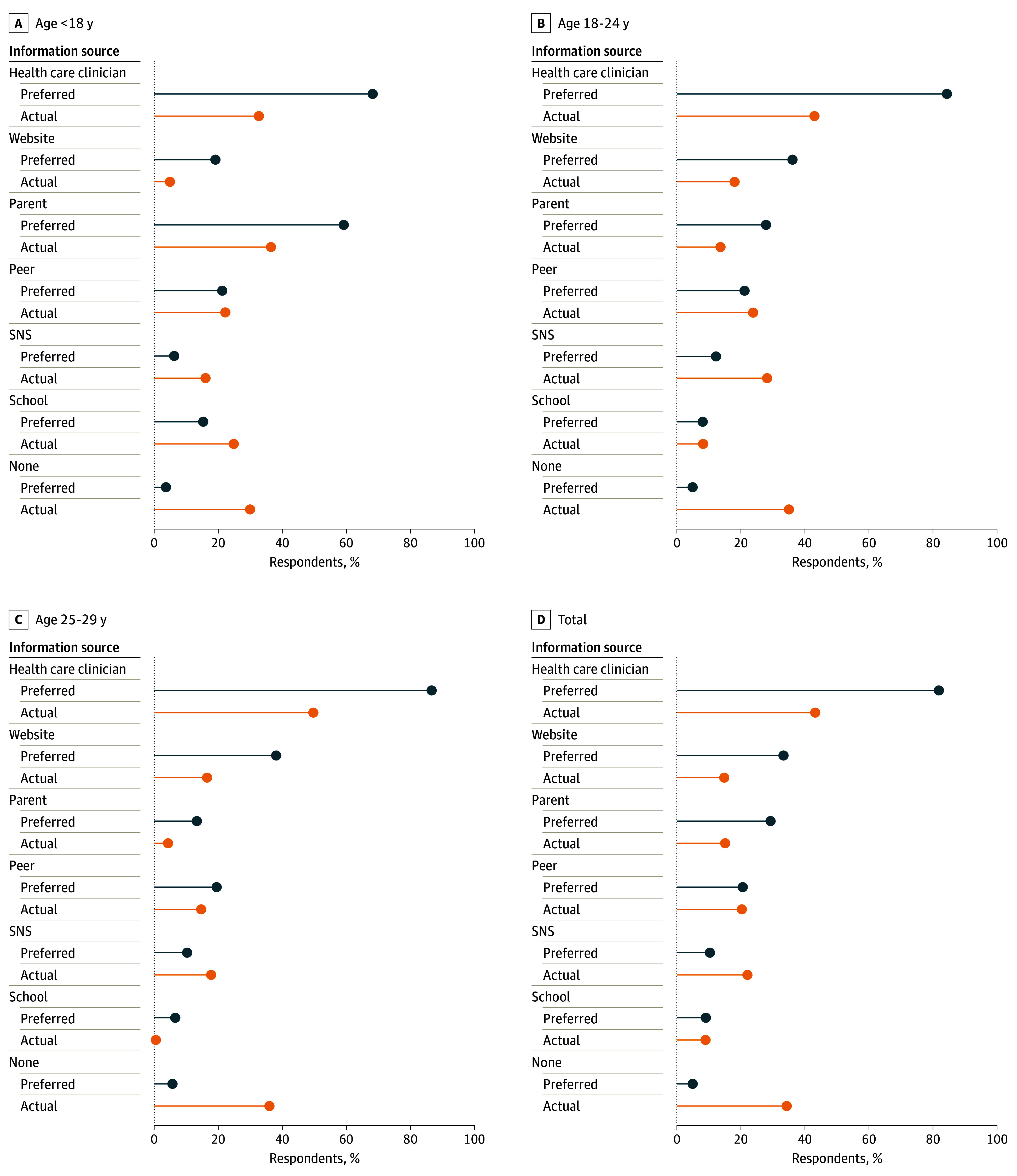
Preferred and Actual Sources of Contraceptive Information Among Thanks, Birth Control Survey Respondents Stratified by Age Group (N = 1150) SNS indicates social networking site.

Participants’ actual source was associated with having self-reported sufficient information about contraception (Table). Participants had greater odds of reporting sufficient information if their source was a clinician compared with other sources or no source. Younger participants (aged <18 years) with parents as an actual source also had greater odds of reporting sufficient information. Schools, peers, websites, and social networking sites were not associated with sufficient information.

**Table.** Unadjusted and Adjusted Logistic Regression Models for Actual Source of Contraceptive Information and Association With Having Sufficient Information to Make Contraceptive Decision (N = 1150)^a^

Actual information source	Enough information to decide which method of contraception is right for you^b^					
	Aged <18 y		Aged 18-24 y		Aged 25-29 y	
	OR (95% CI)^c^	aOR (95% CI)^d^	OR (95% CI)	aOR (95% CI)	OR (95% CI)	aOR (95% CI)
School	0.85 (0.44-1.65)	0.86 (0.44-1.69)	2.02 (0.55-7.38)	2.55 (0.71-9.13)	2.25 (0.21-23.72)	1.39 (0.13-14.88)
Physician, nurse, or other clinician	2.93 (1.51-5.69)	2.77 (1.39-5.53)	3.05 (1.76-5.30)	2.58 (1.48-4.49)	3.72 (2.05-6.77)	3.33 (1.79-6.20)
Parent(s) and/or caregiver(s)	3.61 (1.94-6.73)	3.29 (1.74-6.21)	1.06 (0.49-2.28)	1.03 (0.45-2.37)	1.20 (0.23-6.27)	1.81 (0.39-8.29)
Peers (friends, partner, siblings, other)	1.23 (0.62-2.46)	0.97 (0.48-1.97)	1.50 (0.79-2.84)	1.27 (0.64-2.51)	0.77 (0.39-1.54)	0.74 (0.37-1.47)
A website	0.83 (0.25-2.80)	0.69 (0.19-2.46)	2.08 (0.99-4.36)	1.92 (0.86-4.28)	1.47 (0.68-3.17)	1.16 (0.56-2.43)
Social media, such as Instagram or TikTok	0.65 (0.30-1.42)	0.56 (0.25-1.28)	0.67 (0.38-1.20)	0.60 (0.33-1.10)	0.62 (0.32-1.21)	0.61 (0.29-1.26)

Abbreviations: aOR, adjusted odds ratio; OR, odds ratio.

^a^ All results are weighted. We evaluated statistical significance with α = .05 and used 2-sided hypothesis tests.

^b^ Less than 1% of responses (between 5 and 9 participants) were missing and excluded from the presented regression analysis.

^c^ Reference category includes adolescents and young adults who did not receive information from any sources in the past year.

^d^ Adjusted for whether the respondent has ever had penile-vaginal sex.

## Discussion

This study’s results suggest discrepancies between preferred and actual sources of contraceptive information for AFAB AYAs in the US. Findings underscore the role of clinicians in supporting informed contraceptive decision-making among AYAs. Clinicians were the most commonly preferred source, and receiving information from them was associated with having sufficient information to choose a contraceptive method; however, clinicians were the source with the largest discrepancy between preferred and actual use. Limitations include potential selection bias due to the parental consent requirement for minor participants and recall bias for past year information sources. Findings highlight the continued value of parents and clinicians as sources of contraceptive information and suggest a need to improve information in digital spaces. Clinician engagement in online health education may be one strategy to help AYAs access preferred contraceptive information.

## Notes

Supplement 1. **eAppendix.** Weighted Demographic Characteristics of Thanks, Birth Control Survey Respondents (n=1,150)

Supplement 2.
Data Sharing Statement
