# Supplementary material for: Adolescents and Young Adults’ Sources of Contraceptive Information
Source: JAMA Netw Open. 2024 Sep 13;7(9):e2433310. doi: 10.1001/jamanetworkopen.2024.33310 (PMC11400216; doi:10.1001/jamanetworkopen.2024.33310)
Supplement: Supplement 1. — eAppendix. Weighted Demographic Characteristics of Thanks, Birth Control Survey Respondents (n=1,150) [file jamanetwopen-e2433310-s001.pdf]

Supplemental Online Content

Pleasants E, Whitfield B, Pleasure ZH, et al. Adolescents and young adults' sources of contraceptive information. *JAMA Netw Open.* 2024;7(9):e2433310. doi:10.1001/jamanetworkopen.2024.33310

eAppendix. Weighted Demographic Characteristics of Thanks, Birth Control Survey Respondents (n=1,150)

This supplemental material has been provided by the authors to give readers additional information about their work.

© 2024 Pleasants E et al. *JAMA Network Open.*

eAppendix. Weighted demographic characteristics of Thanks, Birth Control survey respondents (n=1,150)

	**N (mean or %)**
Age	1,150 (22.1)
Age category	
<18	237 (20.6%)
18-24	508 (44.2%)
25-29	405 (35.2%)
Education level	
Currently in high school	232 (20.2%)
Less than HS	85 (7.4%)
HS	262 (22.8%)
Some college	314 (27.3%)
Bachelor or higher	258 (22.5%)
Racialized identity and ethnicity	
Non-Hispanic white	590 (51.3%)
Non-Hispanic Black	162 (14.1%)
Non-Hispanic other	57 (4.9%)
Hispanic	275 (23.9%)
Non-Hispanic two or more races	66 (5.8%)
Ever had penile-vaginal sex	598 (52.8%)
Had penile-vaginal sex in past 30 days	439 (38.2%)

© 2024 Pleasants E et al. *JAMA Network Open.*
