# Supplementary material for: Adolescents and Young Adults’ Sources of Contraceptive Information
Source: JAMA Netw Open. 2024 Sep 13;7(9):e2433310. doi: 10.1001/jamanetworkopen.2024.33310 (PMC11400216; doi:10.1001/jamanetworkopen.2024.33310)
Supplement: Supplement 2. — Data Sharing Statement [file jamanetwopen-e2433310-s002.pdf]

Data Sharing Statement

Pleasants. Adolescents and Young Adults' Sources of Contraceptive Information. *JAMA Netw Open*. Published September 13, 2024. doi:10.1001/jamanetworkopen.2024.33310

Data
Data available: No

Additional Information
Explanation for why data not available: Data are available upon request from Power to Decide, access was obtained for this analysis but data access is not at the discretion of the authors of this submission.
